# An Improved Mixture Density Network for 3D Human Pose Estimation with Ordinal Ranking

**DOI:** 10.3390/s22134987

**Published:** 2022-07-01

**Authors:** Yiqi Wu, Shichao Ma, Dejun Zhang, Weilun Huang, Yilin Chen

**Affiliations:** 1 School of Computer Science, China University of Geosciences, Wuhan 430074, China; wuyq@cug.edu.cn (Y.W.); msc@cug.edu.cn (S.M.); 2 Hubei Key Laboratory of Intelligent Robot (Wuhan Institute of Technology), Wuhan 430205, China; yilinchen@wit.edu.cn; 3 School of Geography and Information Engineering, China University of Geosciences, Wuhan 430074, China; 201920632@cug.edu.cn; 4 School of Computer Science and Engineering, Wuhan Institute of Technology, Wuhan 430205, China

**Keywords:** 3D human pose estimation, mixture density network, Gaussian mixture model, graphic convolutional network, ordinal ranking

## Abstract

Estimating accurate 3D human poses from 2D images remains a challenge due to the lack of explicit depth information in 2D data. This paper proposes an improved mixture density network for 3D human pose estimation called the Locally Connected Mixture Density Network (LCMDN). Instead of conducting direct coordinate regression or providing unimodal estimates per joint, our approach predicts multiple possible hypotheses by the Mixture Density Network (MDN). Our network can be divided into two steps: the 2D joint points are estimated from the input images first; then, the information of human joints correlation is extracted by a feature extractor. After the human pose feature is extracted, multiple pose hypotheses are generated via the hypotheses generator. In addition, to make better use of the relationship between human joints, we introduce the Locally Connected Network (LCN) as a generic formulation to replace the traditional Fully Connected Network (FCN), which is applied to a feature extraction module. Finally, to select the most appropriate 3D pose result, a 3D pose selector based on the ordinal ranking of joints is adopted to score the predicted pose. The LCMDN improves the representation capability and robustness of the original MDN method notably. Experiments are conducted on the Human3.6M and MPII dataset. The average Mean Per Joint Position Error (MPJPE) of our proposed LCMDN reaches 50 mm on the Human3.6M dataset, which is on par or better than the state-of-the-art works. The qualitative results on the MPII dataset show that our network has a strong generalization ability.

## 1. Introduction

With the popularity of virtual reality devices, the task of 3D human pose estimation from a monocular image is gaining importance and becoming one of the challenging tasks in the field of computer vision. A general and effective method for 3D human pose estimation is a two-stage approach, in which the 2D joints are detected from a monocular input first [1], and then, the 3D pose is generated from the detected 2D joints [2,3,4,5,6,7,8,9]. The advantage of the two-stage approach is that it directly predicts a 3D pose from simple 2D joints, diminishing the interference of input RGB images, such as variations in the background scene, lighting, clothing color, etc. In addition, the two-stage approach can fully utilize the 2D joint labels in the human pose dataset and is adapted to both indoor and in the wild scenarios.

While deep learning brings remarkable progress in many computer vision tasks, 3D human pose estimation from a monocular image still faces the problem of depth ambiguity. To be specific, different 3D human poses may have the same or similar 2D projections under different conditions, such as lighting or viewing angle, making the task of estimating 3D human poses based on deep learning from a single RGB image uncertain. Some methods use various geometric constraints between joints, such as joint limits [2] and bone length [10], etc., to eliminate infeasible 3D poses. However, there may still be the problem of multiple 3D poses with similar 2D projections. Some recent approaches generate multiple hypotheses of geometrically feasible 3D human pose to solve the uncertainty problem. Jahangiri and Yuille [11] were the first to propose the idea of generating multiple 3D pose hypotheses.

In contrast to single estimation, it can effectively eliminate model over-fitting and enhance the generalization capability. Based on it, Li et al. [12] proposes a two-stage approach that adopts the Mixture Density Networks (MDN) [13] for the 3D human pose estimation task and achieves state-of-the-art results. The network first lifts the 2D joint into a latent space via a feature extractor only implemented by a simple Fully Connected Network (FCN); then, multiple hypotheses are generated by a hypothesis generator. However, the network has two obvious drawbacks due to its several linear layers. First, a number of learnable parameters that come from dense connections might result in over-fitting. Second, the robustness of this network is insufficient due to the limited feature extraction capability caused by an overly simplistic network structure. In addition, this method outputs multiple results, and the selection of a single result relies on the ground truth label. Hence, the actual estimation results are random and unstable in real-world applications where there is no 3D human pose ground truth label.

In order to solve the problems above, we propose the Locally Connected Mixture Density Network (LCMDN) for 3D human pose estimation. The LCMDN proposes improvements for the MDN [12] and is inspired by the idea of the Locally Connected Network (LCN) [14,15], which introduces a variant of GCN for the 2D to 3D task.

**Figure 1:**
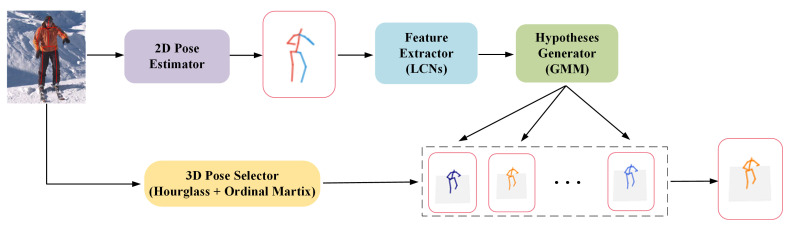
Pipeline for the proposed Locally Connected Mixture Density Network (LCMDN). Our network takes RGB images as input and outputs multiple predictions of 3D pose first. Then, a 3D pose selector is trained to select the best estimation result.

Figure 1 is the pipeline of our proposed LCMDN. Our network can be divided into two steps: the 2D joint information is first obtained from the input image, and then, a feature extractor is used to extract features, and the parameters of a Gaussian mixture model (GMM) is obtained by the hypotheses generator. Multiple predictions of 3D human poses can be generated from the GMM. Specifically, our network uses LCN instead of the simple dense connections in a feature extractor, reducing the challenge of over-fitting due to sparse joint connections between joints. Our model predicts multiple possible hypotheses by MDN so that its robustness is significantly improved. Moreover, we introduce a pose selector to select one pose that best matches the human joint correspondence to solve the problem of ground truth absence in real-world applications.

We test the proposed LCMDN on public datasets. Qualitative experiments show that our method can effectively obtain accurate 3D human poses from 2D images. Quantitative experiments show that our network achieves outstanding estimation accuracy. In addition, we conduct robustness tests and ablation studies for the proposed LCMDN for further analyses of the network’s ability. The main contributions of this paper are listed as follows:

(1) We propose an LCN-based human pose estimation network that learns a Gaussian mixture model matching the distribution of human joints to output multiple hypotheses.
(2) LCN is applied to a 3D human pose estimation task with multiple pose outputs, which improves the accuracy of the estimation task by learning the structural relationships of human joints.
(3) A 3D pose selector is design to select the best predicted 3D human pose. In the selector, an ordinal matrix containing joints relationship is learned from the input RGB images via an hourglass network.
(4) Our network achieves comparable or better results than the state-of-the-art in terms of accuracy and visualization with better robustness, and experimental results on the MPII human dataset validate the generalization ability of our method.

## 2. Related Work

### 2.1. Graph Convolutional Networks

Graph convolution network (GCN) is commonly visible in computer vision and achieves state-of-the-art performance by leveraging GCN to model the relations such as temporal sequences [16,17] and visual objects [18,19]. GCN can be divided into two categories: the spatial domain [20,21,22,23] and the spectral domain [14,24,25]. For the spatial domain, the convolution process is performed with the Fourier transform, while for the latter, the spectral domain is applied directly to the nodes of the graph and their neighbors. Our work belongs to the latter stream, where convolution operates in the spectral domain.

Currently, several works attempt to apply GCN to human pose estimation tasks. Zhao et al. [23] proposed semantic graph convolution and integrated nonlocal layers into the network to expand the acceptance domain of the graph kernel. The LCN [14,15] is proposed to overcome the limitations of the GCN by assigning dedicated rather than shared filters to different joints.

In this paper, we apply the idea of graph convolution to the MDN-based human pose estimation task to replace FCN. Our proposed LCMDN learns the semantic relationships between body joints by introducing a unique weight matrix for each joint point.

### 2.2. 3D Pose Estimation

Existing methods for estimating 3D human pose are divided into two categories according to their inferring process. One is an end-to-end approach [10,26,27,28,29,30,31,32] based on deep convolutional neural networks (CNNs) where 3D human poses are directly generated from the input images. Zhou et al. [28] represent the 3D pose as a sparse representation and predict the 3D pose using an expectation–maximization (EM) algorithm. Park et al. [29] concatenate detected 2D poses and additional information on relative location among multiple joints to improve traditional CNNs. Pavlakos et al. [26] come up with a volumetric representation to predict 3D heatmaps considered as a volumetric version of the stack hourglass network [1]. Yang et al. [30] proposes an adversarial network to separate the ground truth 3D labels from generated labels. Simultaneously, Zhou et al. [10] propose a weakly supervised transfer learning approach by using mixed 2D and 3D annotations where 3D pose labels in indoor environments can be applied to in-the-wild inputs.

The other method [2,3,4,5,6,7,8,9] first predicts 2D pose [1,33] joints from a monocular image followed by lifting 2D to 3D human poses by fitting a probabilistic 3D pose model. The two-stage approach decouples a complicated problem into two easier processes. Akhter et al. [9] estimate the 3D pose from detected 2D joints using a multi-stage approach providing an over-complete dictionary of poses. Bogo et al. [4] optimize the error between the reprojected 3D pose and detected 2D joints. Martinez et al. [5] directly regress a 3D pose from given 2D joints by a simple fully connected residual network. To solve the uncertainty problem from 2D to 3D joints, Jahangiri and Yuille et al. [11] first propose an approach to generate multiple pose estimation hypotheses to solve this problem. The authors first learned from a set of 3D human poses uniformly sampled from a 3D human pose dataset and then learned a 3D GMM model using conditional sampling. Li et al. [12] improved on this by combining the traditional neural network with a mixed density model to solve the depth in 3D human pose estimation blurring and occlusion problems.

Considering the special characteristics of human joints, most of the multi-pose estimation hypothesis methods do not exploit the structural relationships between joints. In view of this, we apply the LCN to the 3D human pose estimation task with multi-pose output and design a 3D human pose estimation network that can exploit the structural connectivity relationships of human joints effectively based on a step-by-step training model.

Moreover, the methods using a mixed density model [12,34] have the shortcomings that the selection of optimal results is unstable in practice, as 3D pose labels are not provided in the real-world applications. To solve this problem, we use the hourglass network to learn the position relationship between the joints from the images and use it as an indicator to select the 3D human pose that best matches the actual results.

## 3. Locally Connected Mixture Density Network

**Figure 2:**
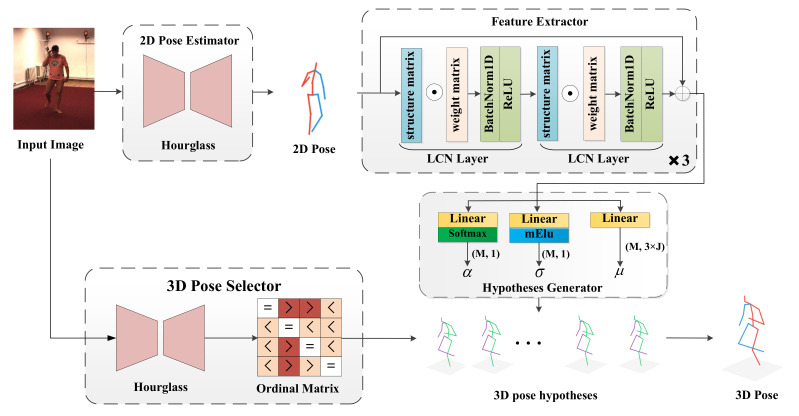
Illustration of the proposed Locally Connected Mixture Density Networks. The network consists of a 2D pose estimator, a feature extractor, a 3D pose hypotheses generator, and a 3D pose selector. The hypotheses generator outputs multiple hypothetical 3D poses from the detected 2D joints. The ordinal matrix is used to generate the ordinal ranking of joints to select the correct human pose estimation. *M* denotes the numbers of Gaussian components.

In this section, we first introduce the modeling of our proposed LCMDN. The architecture of LCMDN is shown in Figure 2. The LCMDN takes the 2D image as input and outputs a correct 3D human pose. The network consists of a 2D pose estimator, feature extractor, hypothesis generator, and 3D pose selector. Details of each module are introduced in the following subsections.

### 3.1. Model Representation

Different from directly training CNNs to estimate the 3D human poses from images or 2D poses, our network can estimate multiple diverse 3D pose hypotheses by learning the Gaussian distribution of human body poses. More specifically, the probability density of 3D pose joints $Y\in R^{3N}$ can be represented as a linear combination of Gaussian kernel functions when inputting the 2D joints $x\in R^{2N}$, which are denoted as

(1) $$p\left(Y|x,\Theta \right)=\sum_{m=1}^{M}\alpha_{m}\left(x,\Theta \right)\phi_{m}\left(y|x,\Theta \right),$$

where Θ represents the learnable parameters of the network, *M* is the number of Gaussian kernels, $\alpha_{m}$ is the mixing coefficient of the *m*th Gaussian distribution, and $\phi_{m}$ represents the *m*th Gaussian distribution, which can be denoted as

(2) $$\phi_{m}\left(y|x,\Theta \right)=\frac{1}{{\left(2\pi \right)}^{c/2}\sigma_{m}{\left(x,\Theta \right)}^{c}}exp\left(-\frac{∥y-\mu_{m}\left(x,\Theta \right)∥{}^{2}}{2\sigma_{m}{\left(x,\Theta \right)}^{2}}\right),$$

where $\mu_{m}$ and $\sigma_{m}$ denote the mean and variance of the *m*th kernel, respectively.

The whole training process of our network can be described as follows: given the 2D joint detections, it outputs the parameters of the Gaussian mixture model. The multiple hypotheses for 3D human pose estimation can be generated from the Gaussian mixture model.

Furthermore, in our proposed LCMDN, to select the best 3D pose from the multiple hypotheses, a joints ordinal ranking matrix is generated from the input image, which indicates the depth position relationship between joints. The estimated pose that matches the relationship best is considered as the final estimated 3D human pose.

### 3.2. Two-Dimensional (2D) Pose Estimator and Feature Extractor

To extract 2D pose joints, the state-of-the-art stacked hourglass network [1] is adopted as the 2D pose estimation module. We use LCN layers to extract more valid information in the feature extractor.

For existing classical MDN-based 3D pose estimation methods [35], the fully connected layers are often adopted for feature extraction. The Laplace operator of FCN and LCN can be transformed into the product of a structure matrix and a weight matrix, which can be formalized as:

(3) y=X(S⊙W),

where structure matrix *S* is shared for all LCN layers and is constructed based on the specified joints dependence. Weight matrix *W* is learned end-to-end, and it varies with different LCN layers.

**Figure 3:**
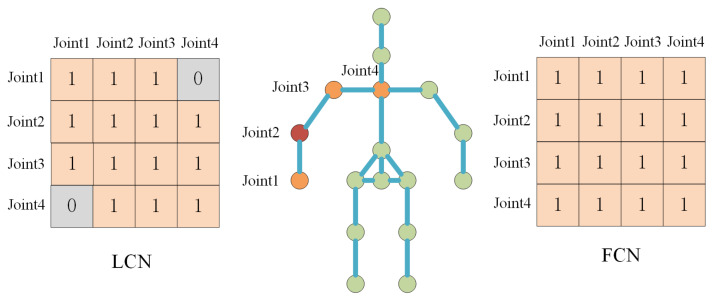
The difference of structure matrix between LCN and GCN. For Joint2, the LCN considers only the relationships within the orange joints, while the FCN considers the relationships with all other joints.

Figure 3 reflects the difference in the structure matrix between FCN and LCN. Taking Joint2 in the figure as an example, FCN considers the relationship between it and all other joints, while LCN only considers associated joints, such as Joint1, Joint3, and Joint4.

Joint dependence is determined by ensuring whether the distance between two joints is less than a hyper-parameter *K*. For instance, the distance between Joint2 and Joint1 is 1 because they are directly connected. The distance from Joint2 to Joint4 is 2 because they are connected indirectly through Joint3.

Specifically, as shown in Figure 2, the feature extractor takes the information of the coordinates of the J joints obtained from the RGB images as input, obtains the feature vectors containing the relationship of the joints through the LCN layers, and projects them to a high-dimensional vector. Each LCN layer also contains the batch normalization layer and activation layer (ReLU). Residual connection is introduced between every two LCN layers to ensure the validity of the features learned by deeper layers.

### 3.3. Hypotheses Generator

Unlike the regression of 3D human joint point coordinates directly from human pose features, the role of the hypotheses generator is to learn the Gaussian mixture distribution model of human 3D joint points from human pose features. As shown in Figure 2, the human pose feature is fed into three different linear connectivity layers and outputs the mixing coefficients α, mean μ and variance σ of the Gaussian mixture model, using three kinds of activation functions, where *M* denotes the number of Gaussian kernels.

Specifically, suppose the human features learned in the feature generator are *x*. $F_{\mu }$, $F_{\mu }$, and $F_{\sigma }$ represent three different linear layers, respectively. The mean value μ reflects the average degree of 3D human joint point information, so the mean value can be calculated by one linear layer, which is denoted as:

(4) $$\mu =F_{\mu }\left(x\right)$$

The mixing coefficients α reflect the weights of the single Gaussian model with a sum of 1, and each value satisfies the range from 0 to 1. Therefore, the softmax function is used as the activation function in the output, which is defined as:

(5) $$\alpha =\frac{F_{\alpha }\left(x\right)}{\sum_{M}^{}F_{\alpha }\left(x\right)}$$

To ensure the validity of the variance σ, the ELU function is chosen as the activation function to calculate the variance. In addition, in order to make the value of the variance always greater than 0, each term of the original formula definition is added by 1. The modified ELU function is defined as function 6, where γ represents a scale for negative factors.

(6) $$\sigma =\left\{\begin{array}{c} F_{\sigma }\left(x\right)+1,F_{\sigma }\left(x\right)\geq 0\\ \gamma \left(exp\left(F_{\sigma }\left(x\right)\right)-1\right)+1,F_{\sigma }\left(x\right)<0 \end{array}\right.$$

### 3.4. 3D Pose Selector

In recent work [36,37,38,39], ordinal relations have been used in 3D human estimation tasks with ordinal annotations to impose penalties for violations of ordinal depth constraints. The 3D pose selector is introduced to select the most realistic 3D pose from the generated multi-pose human hypotheses.

Similar to Sharma [36], we use the ordinal ranking to estimate the depth and reflectance of human joints. Our network uses human RGB images as input and a four-layer stacked hourglass network to obtain the ordinal matrix. As shown in Figure 2, the human joints are listed in rows and columns of the ordinal matrix. The matrix reflects the ordinal relations of each pair of human joints, in which >,=,< represents the positional relationship of less, greater and equal, respectively. See more details of the ordinal matrix in [36].

The whole training process is divided into two parts. Firstly, multiple 3D poses are output from the Hypothesis Generator. Then, the pose selector is used to obtain the ordinal matrix from the input images. The obtained matrix is compared with the ordinal matrix generated from the predicted poses. The one that matches best is used for the backpropagation in the training phase.

During the testing phase, multiple human pose hypotheses are generated, and corresponding ordinal matrices are outputted. Then, the pose selector selects the most realistic 3D pose by comparing ordinal matrices and finding the best match.

## 4. Experiments

In this section, qualitative and quantitative experiments are conducted to evaluate our proposed LCMDN. At first, implementation details, datasets and metrics are presented. Then, we test the network on the Human3.6M [40] and MPII [41] dataset and make comparisons to the state-of-the-art methods. Some additional ablation studies are designed to demonstrate the capability of the network.

### 4.1. Training Details and Developing Environment

For the optimizer, we choose the Adam [42] with exponential decay and set the initial learning rate to 0.001. For the initialization of the network, the Kaiming initialization [43] is applied. The batch size is 64, and the network is trained for 50 epochs. The testing time of our proposed LCMDN from the RGB image to 2D pose is about 78 ms and from 2D pose to 3D pose is about 0.8 ms on average. The max-norm constraint is used to ensure the weight of each layer in [0, 1]. The value of $\alpha_{i}\left(x\right)$ and $\sigma_{i}\left(x\right)$ are fixed to [1 × 10${}^{-8}$, 1] and [1 × 10${}^{-15}$, 1 × 10${}^{15}$], respectively, to maintain the training loss. As a two-stage estimation method, the stacked hourglass network [1] method for 2D joints detection is adopted. We use the MPII dataset for the pre-training of the 2D joints detection network and the Human3.6M dataset for the fine-tuning.

The hardware for experiments includes the following: CPU: Intel i5-10500, GPU: RTX 3060, RAM: 32 GB. The developing environment is Ubuntu16.04, CUDA 10.1, cuDNN 7.4, and TensorFlow.

### 4.2. Dataset and Metric

**Human3.6M dataset [40]:** The Human3.6M dataset is a large public human pose dataset with about 3.6 million indoor images. These images are captured to show 15 different human actions performed by seven people. The dataset offers both 2D and 3D human pose ground truth by labels of human joints’ positions. Different subjects are selected for training (1,5,6,7,8) and testing (9,11). We follow [5] for the standard normalization of both 2D and 3D joints.

**MPII dataset [41]:** The MPII dataset is a small 2D human pose dataset with image data mainly from major video sites, such as YouTube, and it provides before and after unannotated frames. The MPII dataset includes about 25,000 images with human behavioral actions, including over 40,000 annotated human joints, covering 410 human activities, including walking, sitting, skiing, and hiking. It is one of the mainstream human pose datasets. Since the MPII dataset does not provide 3D pose information, we only report qualitative visualization results for the MPII dataset.

**Evaluation Metric:** For Human3.6M, there are two common evaluation protocols. One standard protocol computes the Mean Per Joint Position Error (MPJPE) in millimeters between the ground truth and predictions after being aligned with the root joint. This metric is called Protocol #1. The other protocol further aligns the predictions with the ground truth, leveraging a rigid transformation. This protocol is called Protocol #2. We use the more challenging Protocol #1 for evaluation in all the experiments.

### 4.3. Results on Human3.6M Dataset

The experiment results of our method and several latest methods for 3D pose estimation on the Human3.6M dataset are shown in Table 1. From the table, the LCMDN outperforms other methods in most human actions. It achieves an average error of 50 mm in 15 human behavior actions, which is a decrease of 5.4% compared to the previous best average error of 52.7 mm. For a relatively simple action “Photo”, the error reaches 58.1 mm, which decreases about 5.8% compared to the previous best result of 61.5 mm. Even for some more complex actions, such as “Eating”, the error is reduced from 44.7 to 44.5 mm.

From the results, although the LCMDN is without any multi-view or video information, it still outperforms most other spatial or temporal constraints-based methods due to the extraction of human joints correlation, which indicates the improvement of our model.

Considering some joints are often occluded or missing due to various kinds of interference in realistic scenarios, we simulate the situation of missing joins by randomly dropping one or two limb joints to test our network. The results are reported in Table 2 together with the performance of some existing methods which generate single hypotheses or use CNN-based networks. From the table, the performance is our LCMDN has significantly improved compared to the baseline methods, which further indicates the robustness of our method.

**Figure 4:**
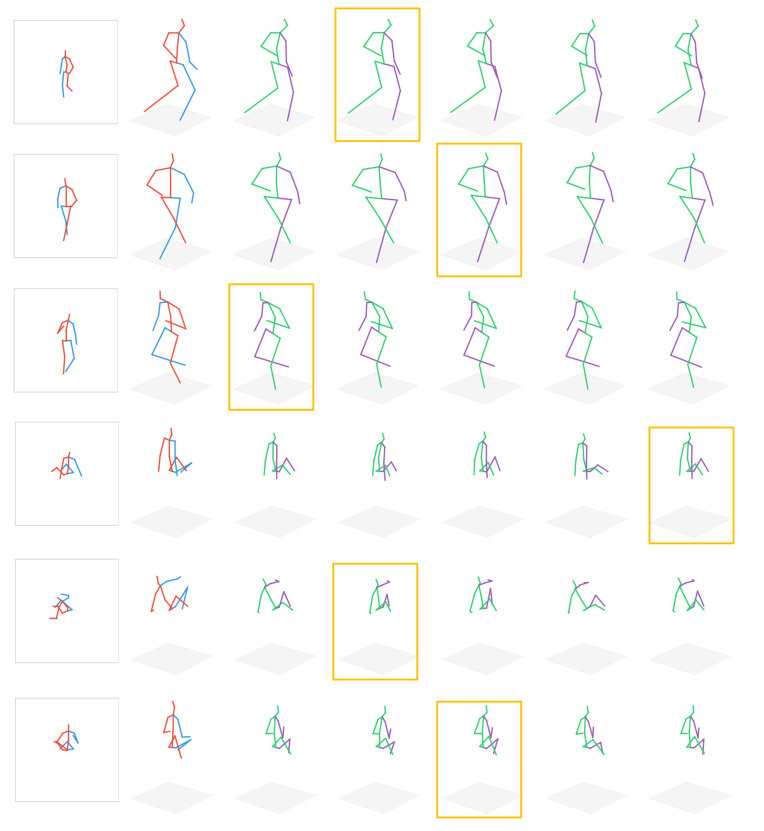
Visualization results on Human3.6M dataset. The first column shows the 2D poses estimated by the stacked hourglass network. The second column shows the ground truth offered by the dataset. The following 5 columns are the estimated hypothetical results generated by the hypotheses generator of the LCMDN, and the poses surrounded by orange frames are the final estimated poses selected by the LCMDN.

To demonstrate the effectiveness of our method more intuitively, we give some visualization results in Figure 4. As can be seen from rows 1 to 3 of the figure, for simple poses with low ambiguity, such as standing, each Gaussian kernel produces almost identical hypotheses, which indicates that a single Gaussian distribution is effective for predicting simple human poses. However, for human poses with more occlusions and ambiguities, such as the sitting pose, different human pose hypotheses are generated, such as the obvious difference between column 5 and column 7 in row 5, which further demonstrates that the multi-pose estimation model can alleviate the uncertainty problem in the single-pose prediction model by generating multiple human pose hypotheses.

### 4.4. Ablation Study

**Different step of neighbors.** To verify the effect of the neighborhood distance *K* (in Section 3.2) on the experimental results, we investigate the value of *K* from 1 to 4. Results and comparisons with LCN [14] on the Human3.6M dataset are shown in Table 3. It indicates that a small value of *K* leads to poorer representation capability of our model. However, too large values also bring the decay of performance due to the redundant features. Therefore, *K* is set to 2 for our network, which ensures that the network can learn valid human joint structure features while reducing the learning of redundant information.

**Different number of kernels.** There are *M* Gaussian kernels in Equation (1), which represent *M* sets of different results generated by the hypotheses generator of our LCMDN. We train four different models with *M* set to 1, 3, 5, and 8, respectively. The results and the comparisons with [12] are shown on the Human3.6M dataset in Table 4. The results show that multiple Gaussian has better performance than single Gaussian. However, when the number of Gaussian kernels is greater than 5, there is only a tiny improvement. In order to balance the computational burden and capability of the network, the number of Gaussian kernels *M* is set to 5 for our network.

### 4.5. Three-Dimensional (3D) Human Pose Estimation on MPII Dataset

More qualitative experiments are conducted on MPII to testify the generalization ability of our LCMDN. The MPII dataset does not offer ground truth 3D labels, so the step of 2D to 3D estimation cannot be trained on it, and the numeric estimation accuracy can not be evaluated. In the experiments, the network settings are the same as experiments in Section 4.3, and we also use the Human3.6M dataset for training.

**Figure 5:**
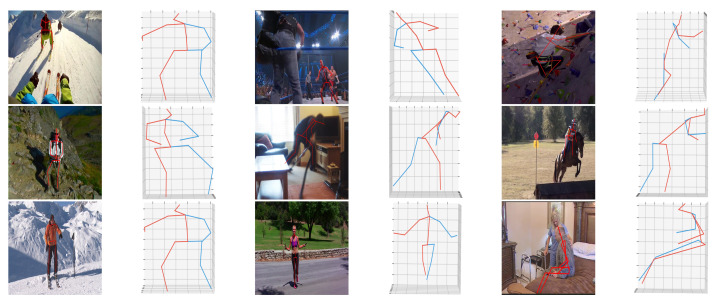
Qualitative results on the MPII dataset. Columns 1, 3, and 5 are the input images and columns 2, 4, and 6 are corresponding 3D poses generated by our network.

Visualization results on the MPII dataset are shown in Figure 5. According to the observation, although the model is trained by the indoor dataset, it also achieves excellent generalization performance to in the wild scenes. Moreover, for actions with serious occlusion problems, such as rock climbing and horseback riding, our LCMDN can also estimate complete and correct 3D human pose. As shown in Figure 5, in the photo of rock climbing (column 5, row 1), although half of the human joints in the image are occluded, the LCMDN can still infer an accurate 3D human pose, which also proves the robustness of the LCMDN.

## 5. Conclusions

In this work, we present a novel network, namely LCMDN (Locally Connected Mixture Density Network), for 3D human pose estimation. Our network introduces the Locally Connected Network to the Mixture Density Network to integrate the representation and optimization ability of these two networks.

The LCMDN generates multiple 3D pose hypotheses at first. Then, the most realistic pose is selected based on the positional relationship of joints via the pose selector of the LCMDN. It solves the result selection problem when there is no ground truth label to evaluate the best result of multiple hypotheses.

Experimental results show that the average MPJPE of our proposed network reaches 50 mm on the Human3.6M dataset, which is on par or better than the state-of-the-art works. Moreover, it is robust in the occlusion or undetected joints scenario and generalizes well for the in the wild scenario. In the future work, we will optimize our network [45,46] for better time performance and apply the idea of LCMDN into more extended fields, such as visual tracking [47], multi-view and multi-person pose estimation [46,48], or 3D human hand pose estimation [49]. 

## Figures and Tables

**Table 1:** The numerical results under protocol # 1 and comparisons on the Human3.6M dataset (mm). (Best result in bold.)

Method	Direct.	Discuss.	Eating	Greet	Phone	Photo	Pose	Purch.	Sitting	SittingD.	Smoke	Wait	WalkD.	Walk	WalkT.	Avg.
Lin et al. [40]	132.7	183.6	132.3	164.4	162.1	205.9	150.6	171.3	151.6	243.0	162.1	170.7	177.1	96.6	127.9	162.1
Du et al. [44]	85.1	112.7	104.9	122.1	139.1	135.9	105.9	166.2	117.5	226.9	120.0	117.7	137.4	99.3	106.5	126.5
Zhou et al. [28]	87.4	109.3	87.1	103.2	116.2	143.3	106.9	99.8	124.5	199.2	107.4	118.1	114.2	79.4	97.7	113.0
Pavlakos et al. [26]	67.4	71.9	66.7	69.1	72.0	77.0	65.0	68.3	83.7	96.5	71.7	65.8	74.9	59.1	63.2	71.9
Jahangiri et al. [11]	63.1	55.9	58.1	64.5	68.7	61.3	55.6	86.1	117.6	71.0	71.2	66.3	57.1	62.5	61.0	68.0
Zhou et al. [10]	54.8	60.7	58.2	71.4	62.0	65.5	53.8	55.6	75.2	111.6	64.1	66.0	51.4	63.2	55.3	64.9
Martinez et al. [5]	51.8	56.2	58.1	59.0	69.5	78.4	55.2	58.1	74.0	94.6	62.3	59.1	65.1	49.5	52.4	62.9
Lee et al. [31]	43.8	51.7	48.8	53.1	**52.2**	74.9	52.7	**44.6**	**56.9**	74.3	56.7	66.4	**47.5**	68.4	45.6	55.8
Li et al. [12]	43.8	48.6	49.1	49.8	57.6	61.5	45.9	48.3	62.0	73.4	54.8	50.6	56.0	43.4	45.5	52.7
Ci et al. [14]	46.8	52.3	44.7	50.4	52.9	68.9	49.6	46.4	60.2	78.9	**51.2**	50.0	54.8	**40.4**	43.3	52.7
**LCMDN**	**42.0**	**47.1**	**44.5**	**48.2**	54.5	**58.1**	**44.0**	45.8	57.9	**71.4**	52.0	**48.7**	52.7	41.3	**42.3**	**50.0**

**Table 2:** Results of missing joints and comparisons. The first three rows show the results of one missing joint, and the last three rows show the results of two missing joints. (Best result in bold.)

Method	Direct.	Discuss.	Eating	Greet	Phone	Photo	Pose	Purch.	Sitting	SittingD.	Smoke	Wait	WalkD.	Walk	WalkT.	Avg.
Jahangiri et al. [11]	108.6	105.9	105.6	109.0	105.5	109.9	102.0	111.3	119.6	107.8	107.1	111.3	108.4	107.0	110.3	108.6
Martinez et al. [5]	57.4	64.6	64.3	65.6	73.3	85.5	61.0	62.1	84.0	101.1	68.2	66.7	70.8	55.6	59.6	69.1
Li et al. [12]	48.9	53.9	54.5	55.5	62.6	70.4	51.3	52.0	69.7	83.9	60.7	57.2	62.4	48.3	50.8	58.8
**LCMDN**	**46.4**	**50.9**	**50.8**	**51.9**	**58.2**	**64.6**	**47.7**	**48.7**	**64.2**	**77.6**	**56.8**	**53.6**	**57.7**	**45.0**	**46.4**	**54.7**
Jahangiri et al. [11]	125.0	121.8	115.1	124.1	116.9	123.8	116.4	119.6	130.8	120.6	118.4	127.1	125.9	121.6	127.6	122.3
Martinez et al. [5]	62.9	66.9	69.9	71.4	80.2	93.8	66.3	65.9	90.6	109.7	74.2	72.1	75.5	61.7	65.7	75.1
Li et al. [12]	54.0	58.5	60.6	61.4	68.6	77.9	56.6	57.0	77.8	92.4	66.2	62.6	67.5	52.5	55.0	64.6
**LCMDN**	**50.3**	**54.8**	**55.3**	**56.8**	**62.9**	**71.4**	**52.5**	**52.4**	**69.6**	**83.6**	**60.7**	**58.2**	**61.9**	**48.8**	**51.6**	**59.4**

**Table 3:** Comparison between different steps of neighbors. The numbers in the first row represent the value of *K*.

Method	1	2	3	4
LCN [14]	58.77	57.73	57.56	58.37
LCMDN	51.28	50.02	50.42	50.45

**Table 4:** Comparison between different numbers of kernels. Numbers in the first row represent the number of kernels.

Method	1	3	5	8
Li et al. [12]	62.9	55.2	52.7	52.6
LCMDN	58.8	52.4	50.0	49.8

## Data Availability

Not applicable.
